# Huntington’s Disease Patient-Derived Astrocytes Display Electrophysiological Impairments and Reduced Neuronal Support

**DOI:** 10.3389/fnins.2019.00669

**Published:** 2019-06-28

**Authors:** Veronica J. Garcia, David J. Rushton, Colton M. Tom, Nicholas D. Allen, Paul J. Kemp, Clive N. Svendsen, Virginia B. Mattis

**Affiliations:** ^1^Board of Governors Regenerative Medicine Institute, Cedars-Sinai Medical Center, Los Angeles, CA, United States; ^2^Divisions of Biomedicine and Neuroscience, School of Biosciences, Cardiff University, Cardiff, United Kingdom

**Keywords:** Huntington’s disease, astrocyte, neuron, iPSC, co-culture, neurodegeneration, HTT, HD

## Abstract

In Huntington’s disease (HD), while the ubiquitously expressed mutant Huntingtin (mtHTT) protein primarily compromises striatal and cortical neurons, glia also undergo disease-contributing alterations. Existing HD models using human induced pluripotent stem cells (iPSCs) have not extensively characterized the role of mtHTT in patient-derived astrocytes. Here physiologically mature astrocytes are generated from HD patient iPSCs. These human astrocytes exhibit hallmark HD phenotypes that occur in mouse models, including impaired inward rectifying K^+^ currents, lengthened spontaneous Ca^2+^ waves and reduced cell membrane capacitance. HD astrocytes in co-culture provided reduced support for the maturation of iPSC-derived neurons. In addition, neurons exposed to chronic glutamate stimulation are not protected by HD astrocytes. This iPSC-based HD model demonstrates the critical effects of mtHTT on human astrocytes, which not only broadens the understanding of disease susceptibility beyond cortical and striatal neurons but also increases potential drug targets.

## Introduction

Huntington’s disease (HD) is a fatal neurodegenerative disease that presents with involuntary motor control as well as cognitive and psychiatric disturbances. HD is directly linked to over 40 CAG trinucleotide repeats in the first exon of the *Huntingtin* gene (Andrew et al., 1993). The number of CAG repeats has a positive correlation between the age-of-onset of symptoms and severity of the disease. When translated, the CAG repeat produces an expanded polyglutamine repeat (polyQ) in the mutant Huntingtin (mtHTT) protein. Though mtHTT is ubiquitously expressed, it primarily leads to dysfunction and progressive death of medium spiny neurons (MSNs) in the striatum, and subsequently cortical projection neurons (Macdonald and Halliday, 2002; Thu et al., 2010; Ross and Tabrizi, 2011). It is still unclear whether this expansion causes a gain of toxic functions, loss of physiological functions of the protein, or both (see Jimenez-Sanchez et al., 2017 for review). Despite knowing for over two decades that expanded *HTT* underlies HD, there is still no successful treatment or comprehensive understanding of the selective neuronal loss.

While neuronal degeneration is central to HD, glial cells also display disease-related phenotypes in HD patients and rodent models (Khakh et al., 2017). Specifically, dysfunctional astrocytes have been shown to play critical roles in the neuronal decline (Khakh and Sofroniew, 2014; Tong et al., 2014; Jiang et al., 2016). HD rodent astrocytes demonstrate impaired glutamate uptake, which increases activation of neuronal NMDA receptors leading to neurotoxicity (Milnerwood et al., 2010). In addition, impaired K^+^ buffering via reduced inward rectifying K^+^ currents causes rapid changes to the excitability of neurons in response to rates of action potential propagation (Tong et al., 2014).

Induced pluripotent stem cells (iPSCs) (Takahashi and Yamanaka, 2006) can be differentiated to provide a disease-relevant source of human neural cell types. Human iPSC-derived HD neurons have been co-cultured with healthy rodent-derived glia, which improve the electrophysiological function and survival of iPSC-derived neurons (Tang et al., 2013). However, the rodent origin prohibits the study of human HD glial phenotypes. Patient-derived iPSCs are a promising cell source to derive mature astrocytes to model their dysfunction in neurodegenerative diseases (Khakh et al., 2017). Co-culture with neurons may enhance the astrocyte maturation process; however, pure astrocytes would be ideal to distinguish the specific role of astrocytes versus neurons in HD. Furthermore, deriving terminally differentiated astrocytes without passaging would reduce the risk of generating reactive astrocytes that could confound disease-specific phenotypes.

In a chimeric mouse model, human HD iPSC-derived glia surrounding non-diseased murine MSNs led to neuronal hyperexcitability and reduced motor performance (Benraiss et al., 2016). In addition, HD patient iPSC-derived astrocytes display autophagocytic vacuoles (Juopperi et al., 2012) and increased evoked inflammatory responses (Hsiao et al., 2014). However, these studies did not assess the functional maturity of the iPSC-derived astrocytes. And, notably, studies to date have yet to investigate whether patient-derived HD astrocytes directly affect human neuronal function.

Here, we present a novel protocol to differentiate patient iPSCs into a pure population of human HD astrocytes. The astrocytes were functionally mature, displaying electrophysiological profiles not typically observed in less mature or stress-activated astrocytes. This included inward rectifying K^+^ currents that were altered in the HD astrocytes, which corroborates previous results in HD (Tong et al., 2014). At early stages in co-culture, HD astrocytes, compared to control astrocytes, provided less support for the maturation of HD and control neurons. Furthermore, the HD astrocytes, compared to controls, did not protect HD or control neurons from toxicity following chronic glutamate exposure. Our data from iPSC-based HD astrocytes recapitulates findings from patient post-mortem samples and HD mouse astrocytes (Khakh et al., 2017), validating this novel use of patient iPSCs to derive pure functional astrocytes. These human astrocytes provide an ideal model to investigate mechanisms of astrocyte–neuron interactions in HD as well as to identify novel therapeutic targets.

## Materials and Methods

### iPSC Lines

The iPSC lines used in this study were purchased from the Induced Pluripotent Stem Cell Core at Cedars-Sinai (Los Angeles, CA, United States). All lines were reprogramed from fibroblast cells. Control lines included CS83iCTR33n (female, age 21, clinically normal); CS00iCTR21n (male, age 6, clinically normal); and CS25iCTR18n (male, 76, clinically normal, brother of affected sibling). HD lines include CS09iHD109n (female, age unknown, clinically affected with 109 and 119 CAG repeats) and CS77iHD77n (male, age unknown, clinically affected with 77 CAG repeats). These lines have tested as karyotypically normal and the CAG repeat lengths measured using automated genotyping on an ABI 3100 (HD iPSC Consortium, 2012).

### Astrocyte Differentiation

All five lines were first differentiated into neural progenitor cells (NPCs) by growing the cells in suspension in media supplemented with epidermal growth factor and fibroblast growth factor (Ebert et al., 2013). The cultures were mechanically propagated as EZ spheres in growth media [70:30 DMEM:F12 (Life Technologies), 2% B27 without vitamin A (Life Technologies), 1% anti-biotic/anti-mycotic (Life Technologies), 100 ng/ml EGF (Peprotech) and FGF (Peprotech)], as described by Ebert et al. (2009). After a minimum of 10 mechanical passages in weekly intervals, the lines were differentiated to astrocytes using the protocol described below.

Stage 1: At between passages 10 and 30, EZ spheres were dissociated to a single cell suspension using TrypLE (Thermo Fisher Scientific). The ratio of live:dead cells was determined using a hemacytometer by Trypan Blue exclusion. The dissociated NPCs were then plated on poly-L-lysine and laminin-coated glass coverslips at a density of 25,000 live cells per 13 mm coverslip in 24-well plates. The cells were seeded in 80 μl of base medium [DMEM:F12 1:1, 2% B27 (Life Technologies) and 1% antibiotic/antimycotic] and cultured overnight in order increase adherence of the cells to the coverslip. The coverslips were then flooded with the Stage 1 astrocyte differentiation medium [DMEM:F12 1:1, 2% B27, 1% antibiotic/antimycotic and 1 μM Arabinosylcytosine (Ara-C; Sigma-Aldrich)]. NPCs were cultured for a week in this medium with half medium changes every 3 days.

Stage 2: To remove the Ara-C after 1 week, the adherent cells were washed twice with base medium before being cultured in the Stage 2 astrocyte medium [DMEM:F12 1:1, 2% B27, 1% antibiotic/antimycotic and 10 ng/ml Lif-1 (Millipore Sigma)] for 1 week, with medium changes every 3 days.

Stage 3: Following 1 week in Stage 2 medium, the cells were dissociated using TrypLE for 5 min to generate a multi-cell suspension lifted from the coverslip. The cells were then washed in base medium and seeded in T25 Nunc tissue culture flasks. The cells were then fed Stage 3 astrocyte differentiation medium [DMEM:F12 1:1, 8% heat inactivated fetal bovine serum (hiFBS), 1% antibiotic/antimyotic and 10 ng/ml Lif-1], which was changed every 5 days. These cells were considered the “*Stage 3*” astrocytes and were used experimentally. Confluent cells were passaged at a ratio of 1:3, typically every 2 weeks. These Stage 3 cells could be frozen for later experimentation or re-plated onto glass coverslips for immediate study (see below).

Stage 4: The final stage of astrocyte differentiation generated the most functionally mature astrocytes. The *Stage 3* cells were dissociated using TrypLE and re-seeded on 13 mm glass coverslips at a density of 50,000 cells per coverslip (or higher, dependent upon the particular iPSC line). Cells were left overnight in *Stage 3* medium before it was changed to *Stage 4* medium [DMEM:F12 1:1, 8% hiFBS, 1% antibiotic/antimyotic, 10 ng/ml Lif-1, 20 ng/ml ciliary neurotrophic factor (CNTF; R&D), 20 ng/ml bone morphogenic protein -4 (BMP-4; R&D) and 5 μM N-[(3,5-Difluorophenyl)acetyl]-L-alanyl-2-phenyl-glycine-1,1-dimethylethyl ester (DAPT; R&D)]. If necessary, the cells were cultured longer in *Stage 3* medium to achieve confluence prior to the final differentiation in *Stage 4* medium, which was important for their survival. After 1 week in this *Stage 4* medium, these cells were considered the mature “*Stage 4*” astrocytes, which were used for the experiments described below.

### Neural Differentiation

iPSC cultures were grown to 70% confluence on Matrigel-coated plates in mTESR medium (Stemcell Technologies). Using a protocol adapted from a previously published ventral forebrain protocol (Telezhkin et al., 2016), iPSCs were switched to SLI medium [Advanced DMEM/F12 (Invitrogen) 1:1 Neurobasal A (Invitrogen), 1% Glutamax (Life Technologies), 2% B27 without vitamin A, 10 μM SB-431542 (Tocris), 1.0 μM LDN-193189 (Stemgent), 1.5 μM IWR1 (Tocris)], and the medium was changed daily. On day 4, 10 μM Y-27632 (Abcam) was added to the cultures 1 h prior to passaging. The cultures were washed with 1X PBS (Corning), treated with accutase (Millipore) for 5 min, agitated to loosen, and centrifuged at 150 x *g* for 3 min. After the supernatant was aspirated, the cells were resuspended in SLI medium containing 10 μM Y-27632 and seeded on a Matrigel-coated plate at a 1:2 density. The cultures were fed daily with SLI medium. On day 8, the cells were passaged 1:2 as described above and seeded onto Matrigel-coated plates in LIA medium [Advanced DMEM/F12 1:1 Neurobasal A, 1% Glutamax, 2% B27 without vitamin A, 200 nM LDN-193189, 1.5 μM IWR1, 20 ng/mL Activin A (R&D)] containing 10 μM Y-27632. The cultures were then fed daily with LIA medium. On day 16, the neural progenitor cells (NPCs) were passaged, as described above, and were re-suspended in plating medium (Advanced DMEM/F12 1:1 Neurobasal A, 1% Glutamax, 2% B27 without vitamin A) at a concentration of 1 x; 10^6^ cells/mL. The neuronal progenitors were then seeded at a density of 25,000 cells per coverslip on either matrigel-coated glass coverslips or glass coverslips previously seeded with astrocytes matured to *Stage 4*, as described below. Excess cells (∼day 16 of differentiation) were cryopreserved in CryoStor freezing medium for use in later experiments.

### Astrocyte Co-culture

*Stage 3* astrocytes were plated and differentiated for 1 week in *Stage 4* medium before seeding the NPCs on top. Dissociated NPCs were plated at a density of 25,000 per coverslip on top of confluent *Stage 4* astrocytes. From this point, only neuronal differentiation/maturation media were used, with no further supplements for the astrocytes.

The neuronal/astrocyte co-cultures were maintained using theSynaptoJuice Protocol (Kemp et al., 2016). During the first week, thecells were differentiated in SynaptoJuice-A medium [Advanced DMEM/F12 1:1 Neurobasal A, 1% Glutamax, 1% antibiotic/antimyotic, 2% B27 without vitamin A, 2 μM PD0332991 (Tocris), 10 μM DAPT, 10 ng/mL BDNF (Miltenyi), 500 nM LM22A4 (Tocris), 10 μM forskolin (Tocris), 3 μM CHIR99021 (Tocris), 300 μM GABA (Tocris), 1.8 mM CaCl_2_ (Sigma), 200 μM ascorbic acid (Sigma)]. SynpatoJuice-A was changed on day 2 and day 5 after plating. On day 7 post-plating, SynaptoJuice-A was replaced with SynaptoJuice-B differentiation/maturation medium (Advanced DMEM/F12 1:1, Neurobasal A, 2% Glutamax, 1% antibiotic/antimyotic, 2% B27, 2 μM PD0332991, 10 ng/mL BDNF, 500 nM LM22A4, 3 μM CHIR99021, 1.8 mM CaCl_2_, 200 μM ascorbic acid). SynaptoJuice-B was changed every 3 days for up to day 24 days post-plating, at which time the cultures were fixed for immunocytochemistry.

### Immunocytochemistry

Immunocytochemistry was performed on poly-ornithine coated 13 mm glass coverslips in 24 well tissue culture plates. Cells were fixed with 4% paraformaldehyde in phosphate buffered saline (PBS) for 10 min at room temperature and then rinsed 3 times for 3 min with PBS. Cells were blocked in 10% bovine serum albumin and 1% TritonX-100 for 1 h at room temperature and then incubated overnight at 4^o^C with antibodies diluted in blocking solution. The following antibodies were used: GFAP (rat, Agilent Technologies Cat# Z0334, RRID:AB_10013382; dilution 1:2500), S100β (mouse, Sigma-Aldrich Cat# S2532, RRID:AB_477499; 1:1000), β3-tubulin/TuJ1 (rabbit, Sigma-Aldrich Cat# T2200, RRID:AB_262133; 1:1000), and gamma aminobutyric acid (GABA; rabbit, Sigma-Aldrich Cat# A2052, RRID:AB_477652; 1:500). Cells were then washed 3 times and incubated for 1 h at room temperature in green and red fluorescent-conjugated secondary antibodies (Alexa Fluor 448 and Alexa Fluor 594, respectively), diluted at 1:500 in blocking solution with 1% normalized serum from the host species of the secondary antibodies. Cells were then washed 3 times, and incubated with the fluorescent nuclear stain, DAPI, followed by two washes. The coverslip was then mounted on a glass microscope slide using fluoromount-G.

### Whole-Cell Patch Clamp

Whole-cell patch clamp was performed on *Stage 3* and *Stage 4* astrocyte cultures. Glass micropipettes were pulled using a Sutter Instruments P-1000 from thin wall borosilicate glass capillaries (World precision instruments) with a tip resistance of between 4 and 6 MΩ. Cells were visualized using a Leica DM-LED microscope. Voltage and current clamp recordings were performed using a Molecular Devices Multiclamp 700B amplifier, Digidata 1300 converter, and PClamp 10 acquisition software (Molecular Devices). Astrocytes with an access resistance above 35 MΩ were excluded. Cells were bathed in an extracellular solution (ECS) containing (in mM): 135 NaCl, 5 KCl, 5 HEPES, 10 Glucose, 1.2 MgCl_2_, and 1.25 CaCl_2_. The internal solution comprised (in mM): 10 NaCl, 117 KCl, 11 HEPES, 2 Na.ATP, 2 MgCl_2_ and 1 CaCl_2_. The cell’s capacitance and access resistance was measured using a 10 mV test pulse from a holding voltage of −70 mV. Channel blockers were perfused with a rapid solution changer (BioLogic Science Instruments).

A voltage-step protocol was used both with and without online leak subtraction to record voltage-gated Na^+^ (Na_*V*_), voltage-gated K^+^ (K_*V*_), inward rectifying K^+^ (K_*ir*_) and tandem pore potassium (K_2P_) currents. Starting from a holding voltage of −70 mV, a 100 ms voltage-step was applied, starting at −120 mV and increasing in 10 mV increments to +40 mV. Na_*V*_ currents were observed as rapidly activating and inactivating inward currents immediately following the start of voltage-steps above approximately −40 mV, and were sensitive to 0.5 μM Tetrodotoxin (TTX; Tocris). K_*V*_ currents were observed as more slowly activating outward currents at voltages more positive than −20 mV which did not completely inactivate during the 100 ms step and were sensitive to 10 mM Tetraethylammonium chloride (TEA; Tocris). K_*ir*_ currents were observed as a persistent inward current at voltage steps below approximately −80 mV and were sensitive to 10 μM Nortriptyline- and Ba^2+^-containing ECS [in mM: 95 NaCl, 5 KCl, 5 HEPES, 10 Glucose, 1.2 MgCl_2_, 27 BaCl_2_ (Sigma-Alrich)_,_ 0.01 Nortriptyline hydrochloride (Sigma-Aldrich)]. K_2P_ currents were only observable in the absence of leak subtraction, and were a non-linear, Goldman-Hodgkin-Katz rectifying current which was insensitive to 10 μM TEA, but sensitive to 10 μM Quinidine (Sigma-Aldrich).

Resting membrane potential (RMP) was assessed in current clamp by averaging from continuous voltage recordings with a 0 pA holding current. Input resistance was measured using 200 ms current-steps, starting at +10 pA and increasing in 10 pA increments to +180 pA. The input resistance was then calculated using Ohms law.

### Microelectrode Array

Astrocytes at *Stage 3* were thawed into flasks and grown for a week before passage. HD (CS09iHD109n1) and control (CTR; CS83iCTR33n2) astrocytes were then seeded onto 48-well transparent microelectrode array (MEA) plates (Axion Biosystems) at between 25 and 200 thousand cells per well. Astrocytes were allowed up to 2 days in *Stage 3* media after seeding before switching to *Stage 4* media for maturation. After 1 week in maturation, control and HD neural progenitors (∼day 16 of differentiation) were seeded on top of the astrocytes at between 50 and 400 thousand cells per well and the co-culture experiment was considered at Day 0. Daily recordings of activity were measured using the Maestro MEA platform and recording software (Axion Biosystems) for 5 min durations. Wave form events were identified using adaptive spike threshold crossing with a standard deviation of noise set at 5.5, and then further validated and sorted to individual neurons using Offline Sorter (Plexon). A minimum of 1 spike per min was used to include neuronal waveforms for analysis, and PCA analysis of each unit cluster was used to exclude noise artifacts. Data was compiled in excel and statistics run using GraphPad Prism 7.04 and SigmaPlot software.

### ELISA

A standard dilution curve (500 to 7.8 μg/mL) was generated by serial dilution of human thrombospondin-1, with a negative control of assay diluent. Each astrocyte-conditioned medium sample was assayed at full and half concentration, diluted in base medium, and additionally the base medium was sampled as a negative control. Thrombospondin-1 was quantified in astrocyte-conditioned medium using a human thrombospondin-1 Quantikine ELISA Kit (R&D Systems), following the manufacturer’s instructions. The plate was read at an excitation wavelength of 450 nm, simultaneously correcting for a 570 nm background signal. The generated standard curve was then used to calculate the concentration of thrombospondin-1 in astrocyte conditioned medium.

### Cell Counts

Fluorescent imaging of astrocyte cultures was performed on a Leica microscope, model DM6000 B. Images were taken from DAPI (400 nm), GFP (470 nm), Texas red (560 nm), and Y5 (620 nm) channels using 10x; and 20x; air objectives. Images were acquired using Leica Application Suite X (Leica Microsystems). Several random and representative images were taken per coverslip and manually counted using ImageJ. Cell bodies, or appropriate cell regions staining positive for cell markers, were counted and expressed as a ratio of the total DAPI-positive cell nuclei counts.

Cells in the neuronal cultures were noted to have much smaller nuclei compared to those in the astrocyte cultures. Nuclei were measured after 16 days of glial differentiation followed by 6 days in neuronal media with or without co-culture. Nuclei size was measured with the Zeiss stereological software nucleator feature using 8 rays, 1 marker per interval. Neuronal cultures and neuronal-astrocyte co-cultures were quantified (at 6 days post-plating) with Zeiss stereological investigator software using a 20x air objective. Cells in the co-cultures were divided into two groups based upon their nuclei size as a means of distinguishing whether they originated from the neuronal or astrocyte differentiation protocols.

### Ca^2+^ Imaging

Six days post-co-culture plating and maintenance in neuronal media, HD (CS09iHD109n1) and control (CS83iCTR33n2) cells were incubated with 5 μM Fluo-3-AM (Fluo-3) for 30 min in their culture medium at 37°C. Excess Fluo-3 was then washed out and replaced with fresh media. Fluo-3 was excited at 490 nm and emission was recorded at 530 nm using a Leica microscope model DM6000 B equipped with a DFC360FX CCD camera. Excitation exposure was between 25 to 35 ms with a gain between 5 and 8x, depending upon region of interest with an acquisition rate of 15.5 Hz. Fluorescence intensity was measured over time in several regions of interest on both astrocytes and neurons using ImageJ software. Off-line subtraction was performed by measuring fluorescence in a region of interest which did not contain cells or cell extensions. Astrocyte- or neuron-dependent events were discriminated by correlating the cell morphology (using transmitted light) with duration of Ca^2+^ response, and further confirmed by the addition of TTX to block Na_*v*_-dependent action potentials in neurons.

### Glutamate Response and LDH Activity

For each of three experiments, four assays were performed, beginning on day 1 after transition to SynaptoJuice B, and each assay was separated by 4–5 days over the 16-day culture duration. Spontaneous MEA recordings were taken up to 1 h before addition of glutamate. Glutamate (100 μM) was then added to each well of the 48-well plate and the neural activity was immediately recorded (note: Advanced DMEM/F12 contains 0.05 mM glutamate). The plate was then incubated for 12 h at 37°C, followed by a recording of activity of the neurons under chronic glutamate exposure. Media was sampled from every well and pooled by cell group (control astrocytes alone, HD astrocytes alone, control astrocytes with control neurons or HD neurons, and HD astrocytes with control neurons or HD neurons). The concentration of lactate dehydrogenase (LDH) was then measured using a Pierce LDH cytotoxicity assay kit (Thermo Fisher Scientific) per the manufacturer’s protocol.

### Statistics

Chi Squared tests were performed on data with a categorical or binomial dependent variable. *T*-tests were performed on data with a continuous dependent variable, following inspection to be sure that the data were normally distributed around each hypothesized mean. Depending on the distribution of the data set, either a parametric or non-parametric analysis of variance was performed on data sets with more than two groups. Nuclei sizes were compared with a one-way ANOVA followed by a Bonferroni post-test. Outliers were excluded from MEA data of spontaneous activity if they exceeded ±2 standard deviations from the mean of three experiments. *Post hoc* tests were then chosen according to the data sets and are stated. Unless stated otherwise, continuous data are reported as mean ± standard error of the mean.

## Results

### Human iPSCs Provide a Pure Population of Astrocytes That Produce Hallmark Proteins

**FIGURE 1:**
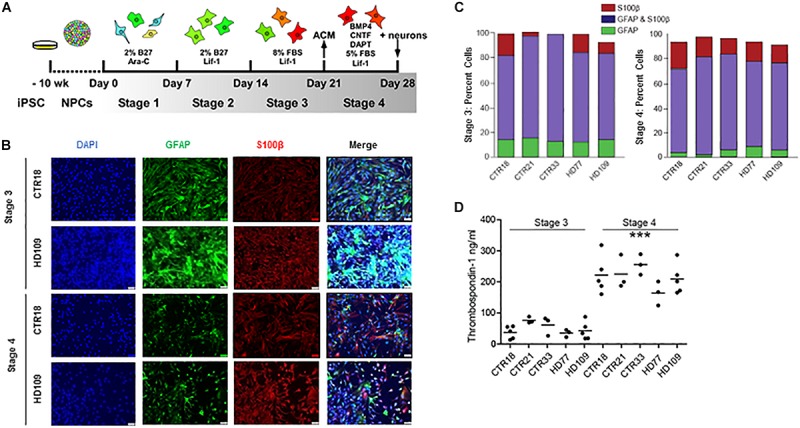
Huntington’s disease and control iPSCs are equally capable of generating mature astrocytes. **(A)** Schematic of astrocyte differentiation from iPSC-derived neurospheres. **(B)** Immunocytochemistry of *Stages 3* and *4* astrocytes stained for GFAP and S100β. **(C)** *Stage 3* (left) and *Stage 4* (right) display astrocyte markers GFAP and/or S100β in equal percentages between HD and controls. (*n* = at least three independent experiments per line and more than 1,900 DAPI-positive cells). **(D)** *Stage 4* astrocytes derived from either HD or control iPSCs release more TSP1 than *Stage 3* astrocytes in all lines tested, indicative of increased maturity (^∗∗∗^*p* < 0.001; *n* = 3–5 independent differentiations).

To examine the influence of HD on astrocytes, astrocytes were differentiated from previously described control (18, 21, or 33 CAG repeats) and HD patient (77 or 109 CAG repeats) iPSC lines (HD iPSC Consortium, 2012). The differentiation protocol consisted of four separate, week-long “stages” that were specifically chosen to rapidly generate mature astrocytes (Figure 1A). In *Stage 1*, iPSC-derived NPCs were treated with 1 μM Ara-C, which inhibits cell division. This was used as neural progenitors require cell division for neurogenesis (Nelson et al., 2008), thereby low doses of Ara-C were used to block neuronal generation. Compared to untreated NPCs, cultures treated with Ara-C contained little to no TuJ-positive neurons (Supplementary Figure 1). In *Stage 2*, Ara-C was replaced by Lif-1 to induce astrogliogenesis (Wright et al., 2003). In *Stage 3*, cells were switched into a Lif-1 and high fetal bovine serum (FBS)-containing medium to elicit astrocyte proliferation. Finally, in *Stage 4*, cells were switched into medium containing further astrogliogenic factors (BMP-4 and CNTF), as well as a γ-secretase inhibitor (DAPT) to encourage synchronous terminal differentiation and maturation (Telezhkin et al., 2016).

Immunocytochemistry assessment of *Stages 3* and *4* astrocytes, from both control and HD iPSC lines, showed a typical large filamentous astrocyte morphology as well as expression of the known astrocyte markers glial fibrillary acidic protein (GFAP) and S100β (Figure 1B). The relative expression of these two proteins can indicate the degree of astrocyte maturation, with GFAP expression present in neural precursors and S100β expression present in more mature astrocytes (Raponi et al., 2007). Quantifying the percent of total cells expressing astrocyte markers showed that *Stages 3* and *4* cultures had a high percentage of both GFAP^+^ and/or S100β^+^ cells (Figure 1C). During the transition from *Stage 3* to *Stage 4*, both control and HD lines had a significant loss of GFAP-positive/ S100β-negative cells (*p* < 0.001; Figure 1C). HD and control cells both expressed markers of mature astrocytes in similar proportions at *Stages 3* and *4*.

The secretion of neurogenic and synaptogenic factors by astrocytes is critical to their function, and pertinent for co-culture studies using astrocytes to enhance neuronal differentiation. Thombospondin-1 (TSP1) is one such synaptogenic factor generated by astrocytes in the developing central nervous system (Chung et al., 2015). To determine the relative functionality of iPSC-derived astrocytes, a TSP1 ELISA was performed on medium conditioned by astrocytes for 24 h. Results revealed a significant increase in TSP1 secretion from *Stage 3* to *Stage 4* (*p* < 0.001; Figure 1D), which was not due to cell number differences as subsequent cell fixation and counts confirmed similar cell numbers between *Stages 3* and *4* cultures (data not shown). The increased TSP1 secretion from *Stage 4* astrocytes supports that the final differentiation step generates functionally mature astrocytes. Similar GFAP and S100β protein production, HD and control astrocytes in *Stages 3* and *4* have no difference in TSP1 levels.

### Human iPSCs Provide a Range of Functionally Mature Astrocytes

In addition to protein expression (Raponi et al., 2007), glial fate can be validated by specific electrophysiological characteristics. Functional traits are cell-specific and demonstrate quantifiable ontogenies during development through to maturity (Shelton and McCarthy, 1999; Zhou et al., 2006). Specifically, immature astrocytes display significant K_*v*_ currents and even small Na_*v*_ currents, reminiscent of very immature neurons (Zhou et al., 2006). As astrocytes mature, they express progressively larger K_*ir*_ and K_2P_ currents (Higashi et al., 2001; Zhou et al., 2006).

**FIGURE 2:**
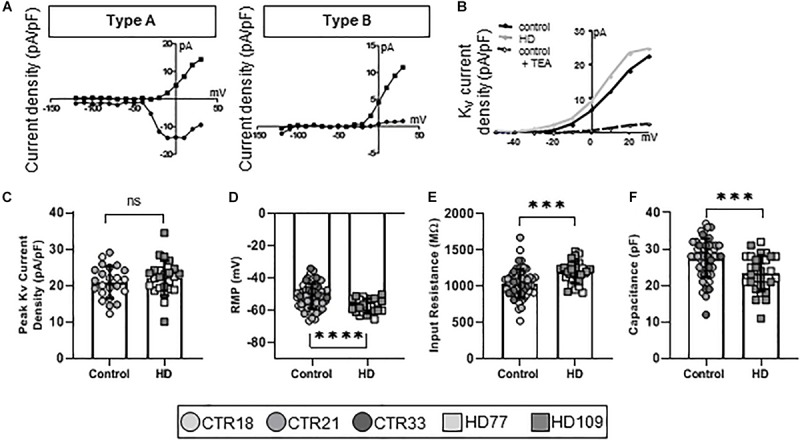
*Stages 3* and *4* HD iPSC-derived astrocytes display reduced capacitance as committed but immature Type A/B astrocytes. **(A)** iPSC-derived astrocytes mature from astrocyte progenitors expressing voltage-gated Na^+^ and K^+^ currents (Type A, left), to immature astrocytes expressing some voltage-gated Na^+^ but mostly voltage-gated K^+^ currents (Type B, right). **(B)** Example current-voltage (IV) curve of voltage-gated K^+^ current density from HD, control and control + TEA in Type A/B astrocytes. **(C)** Immature astrocytes display no differences in the peak voltage-gated K^+^ current density between HD and control. **(D)** Type A/B HD astrocytes have more hyperpolarized resting membrane potentials compared to control and **(E)** higher input resistance. **(F)** Immature HD astrocytes show smaller capacitances compared to controls. Each cell is represented as a single point, and therefore N is represented on each graph. These cells were analyzed from more than three separate experiments (^∗∗∗^*p* < 0.005 and ^****^p < 0.001).

To establish the functional maturity of the *Stages 3* and *4* astrocytes, we employed whole-cell patch clamp electrophysiology to measure ionic currents that are fundamental to astrocyte function. Four electrophysiological types of astrocytes were identified in our cultures and were termed Types A through D. Type A (Figure 2A, left) astrocytes were characterized as having both K_*v*_ and Na_*v*_ currents. Highly immature astrocytes and astrocyte precursors generate very small voltage-gated K^+^ and, if present, voltage-gated Na^+^ currents (Zhong et al., 2016). While this suggests immature neurons, we have shown that these cells are almost entirely GFAP^+^ and/or S100β^+^ (Figure 1B) and have little to no observed TuJ1 staining (Supplementary Figure 1). Collectively, these phenotypes suggest that Type A cells are precursor astrocytes rather than neurogenic. Type B astrocytes expressed only K_*v*_ currents (Figure 2A, right), also indicative of an immature astrocyte progenitor. Pharmacological analysis of the ion channels in Type A and B astrocytes demonstrated the presence of Tetraethylammonium chloride (TEA)-sensitive Kv currents (Figure 2B), as would be expected for a progenitor (Lai et al., 2010). When examining the K^+^ current densities between the immature (Type A and B) control and HD astrocytes, no differences were observed (CTR: 20.93 ± 0.92 pA/pF, HD: 22.12 ± 0.98 pA/pF, *n* = 23 CTR, 24 HD; Figure 2C). To further investigate any HD-related differences in this population of immature astrocytes, we next examined their passive membrane properties. The resting membrane potential (RMP) in glia is important for the control of cell excitability, and contributes to the buffering of extracellular K^+^ and uptake of neurotransmitters (McKhann et al., 1997). The RMP was lower in HD compared to control (CTR: −51.24 ± 1.0 mV, HD: −57.30 ± 0.90 mV, *n* = 56 CTR, 24 HD; *p* < 0.0001; Figure 2D). Input resistance, a crude measure of the concentration of ion channels present on the cell surface, was higher in the HD astrocytes (CTR: 1036 ± 26.18 MΩ, HD: 1188 ± 27.49 MΩ, *n* = 56 CTR, 30 HD; *p* = 0.0001; Figure 2E). And cell capacitance, a precise measure of the cell surface area, was smaller in HD lines compared to controls (CTR: 27.57 ± 0.71 pF, HD: 23.40 ± 0.90 pF, *n* = 54 CTR, 30 HD; *p* = 0.0007; Figure 2F).

**FIGURE 3:**
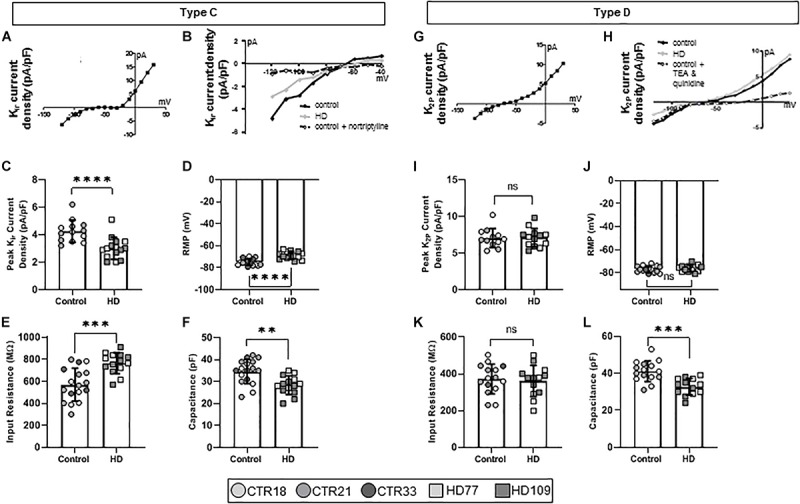
Huntington’s disease iPSC-derived K_*ir*_ channel-expressing (Type C) astrocytes display different current densities, resting membrane potential, input resistance and capacitance. **(A)** Type C astrocytes express K_*ir*_ currents in *Stages 3* and *4* cultures. **(B)** Example IV curve from Type C HD, control and control + nortriptyline astrocytes. **(C)** Type C HD astrocytes display significantly less peak K_*ir*_ current density as compared to controls. **(D)** Type C HD astrocytes have depolarized resting membrane potentials and **(E)** an increased input resistance compared to controls. **(F)** Type C HD astrocytes have decreased capacitance compared to controls. **(G)** Mature Type D astrocytes express K_2P_ currents in *Stages 3* and *4* cultures. **(H)** Example IV curve of K^+^ current density from Type D HD, control and control + TEA and quinidine astrocytes. **(I)** Peak K_2P_ current density, **(J)** resting membrane potential, and **(K)** input resistance are similar between HD and control Type D astrocytes. **(L)** Type D astrocytes have smaller capacitances than control. Each cell is represented as a single point, and therefore N is represented on each graph. These cells all came from more than three separate experiments (^∗∗^*p* < 0.01; ^∗∗∗^*p* < 0.005; and ^****^p < 0.0001).

Over the course of astrocyte development and maturation, K_*ir*_ channels are expressed (Seifert et al., 2009), with K_*ir*_-positive astrocytes present at higher concentrations in the developing brain. Corroborating this *in vivo* phenotype, another electrophysiological phenotype present in the *Stage 3/4* cultures, termed Type C astrocytes, expressed K_*ir*_ currents (Figure 3A). Furthermore, pharmacological analysis showed that Type C astrocytes expressed TEA-resistant, nortriptyline-sensitive K_*ir*_4.1 channels (Figure 3B). While Type A/B astrocytes had similar K_*V*_ current densities between HD and control lines, Type C HD astrocytes expressed smaller peak K_*ir*_ currents compared to controls (CTR: 4.25 ± 0.22 pA/pF, HD: 3.01 ± 0.21 pA/pF, *n* = 13 CTR, 15 HD; *p* < 0.0001; Figure 3C). HD Type C astrocytes also differed in passive membrane properties, including depolarized RMP values (CTR: −74.93 ± 0.70 mV, HD: −68.82 ± 0.93 mV, *n* = 14 CTR, 14 HD; *p* < 0.0001; Figure 3D), greater input resistances for HD astrocytes (CTR: 570.20 ± 36.27 MΩ, HD: 765.7 ± 25.2 MΩ, *n* = 17 CTR, 14 HD; *p* = 0.0001; Figure 3E) and less capacitance (CTR: 34.59 ± 1.34 pF, HD: 28.36 ± 1.14 pF, *n* = 17 CTR, 14 HD; *p* = 0.0014; Figure 3F) compared to control lines.

Astrocytes within the adult brain predominantly display large K_2P_ currents (Seifert et al., 2009), which were identified in the final electrophysiological phenotype present in the *Stage 3/4* cultures, termed Type D astrocytes (Figure 3G). iPSC-derived Type D astrocytes expressed quinidine-sensitive K_2P_ channels that indicate a mature astrocytic fate (Figure 3H). These astrocytes did not demonstrate any difference in the peak K^+^ current density between HD and control lines (CTR: 7.06 ± 0.38 pA/pF, HD: 7.01 ± 0.35 pA/pF, *n* = 12 CTR, 13 HD; Figure 3I). Similarly, passive membrane properties such as RMP (CTR: −77.07 ± 0.73 mV, HD: −76.29 ± 0.83 mV, *n* = 15 CTR, 13 HD; Figure 3J) and input resistance (CTR: 372.50 ± 20.78 MΩ, HD: 361.20 ± 23.50 MΩ, *n* = 12 CTR, 13 HD; Figure 3K) were not different between HD and control lines. However, HD Type D astrocytes had lower capacitance values compared to controls (CTR: 41.13 ± 1.44 pF, HD: 32.81 ± 1.25 pF, *n* = 15 CTR, 13 HD; *p* = 0.0002; Figure 3L).

Collectively, these results demonstrate that this new protocol can differentiate both control and HD iPSC lines into a population of primarily pure astrocytes, and that these astrocytes vary in maturity levels that can be characterized by different electrophysiological properties. Astrocytes “Type A-D” are indicative of increasing maturity levels and, indeed, a detailed analysis of passive properties showed differences between the astrocyte Types driven by maturation (Supplementary Figure 2). While in stage 3 media, only Type A (34.4 ± 8.9% total cells) and Type B (65.6 ± 8.9%) astrocytes were observed (Supplementary Figure 2A). However, when cultured in the final, stage 4 media which promotes functional maturation of the astrocytes, a significant drop in Type A astrocytes was observed (3.2 ± 3.1% total cells), while the proportion of mature Type C and D astrocytes increased (Type B: 39.4 ± 2.73%, Type C: 31.2 ± 3.57%; Type D: 26.0 ± 1.92% total cells; Supplementary Figure 2A). Type C and D astrocytes demonstrated hyperpolarized RMP (Type A&B: −53.10 ± 2.25 mV, Type C: −72.41 ± 1.53 mV, Type D: −77.10 ± 0.61 mV, *n* = 1 mean value per line, 5 lines; *p* < 0.0001; Supplementary Figure 2B), decreased input resistance (Type A&B: 1090 ± 49.76 MΩ, Type C: 651.40 ± 48.94 MΩ, Type D: 369.50 ± 9.51 MΩ; *p* < 0.0001; Supplementary Figure 2C) and larger cell capacitance (Type A&B: 25.65 ± 1.219 pF, Type C: 32.12 ± 1.674 pF, Type D: 38.99 ± 3.08 pF; Type D vs. Type A&B *p* = 0.0028; Supplementary Figure 2D) when compared to the immature Type A/B astrocytes. Additionally, the most mature Type D astrocyte had a lower input resistance compared to Type C astrocytes (*p* = 0.0011). This maturation in electrophysiological properties was conserved across HD and control astrocytes.

### HD Astrocytes Are Less Supportive of Neuronal Spontaneous Electrophysiological Activity Than Control Astrocytes as Evidenced by Ca^2+^ Imaging Experiments

**FIGURE 4:**
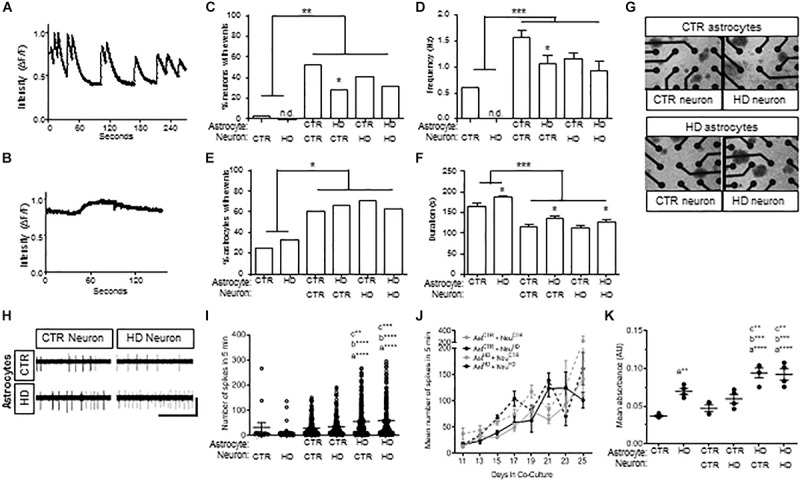
Co-cultures of HD (HD109) and control (CTR33) astrocytes and neurons demonstrate alteration in neuronal firing at early time points and increased toxicity in response to glutamate. **(A)** Example traces of calcium events in control neurons with control astrocytes. **(B)** Example traces of calcium events in control astrocytes with control neurons. **(C)** Percentage of neurons with calcium events is higher in co-culture, but lower in HD astrocyte co-culture compared to control astrocyte co-culture. **(D)** Frequency of events is higher in neurons in co-culture compared to mono-culture, but less frequent in HD astrocyte co-culture compared to control astrocyte co-culture. **(E)** Percentage of astrocytes with calcium transients is greater in co-culture compared to mono-culture. **(F)** Astrocytes in mono-culture had longer transients than in co-culture, and HD astrocytes in mono-culture had longer transients than control astrocytes. Co-culture of control or HD neurons with HD astrocytes showed a longer duration of calcium transients compared to co-culture with control astrocytes. **(G)** Representative images of astrocyte and neuron co-cultures around the 16 electrodes (black circles) of a 48-well MEA plate. **(H)** Example electrode traces showing spikes detected from control (CTR) or HD neurons that are co-cultured with CTR or HD astrocytes. Scale bar: 12.5 μV vertical by 5 s horizontal. **(I)** Spontaneous activity from control and HD co-cultures. Significance between conditions denoted as “a”: from Neu^HD^, “b”: from Neu^CTR^ + Ast^CTR^, “c”: from Ast^CTR^ + Neu^HD^. **(J)** No difference in total mean spike number per neuron in co-culture conditions during daily 5 min recordings over the course of 16 days. Data is presented as separated by the neuron co-culture (gray lines, control neurons; black lines, HD neurons), with solid lines representing control astrocytes and dashed lines indicating HD astrocytes. **(K)** Mean absorbance values of LDH activity in media after 12 h incubation with 100 μM glutamate at 4 time points over 16 days. Significance between conditions denoted as “a”: from Ast^CTR^, “b”: from Ast^CTR^ + Neur^CTR^, “c”: from Ast^CTR^ + Neu^HD^. Ast, astrocyte; Neu, neuron; CTR, control. Calcium imaging: cells were analyzed from more than three separate experiments using a Chi squared test. MEA: activity was analyzed using Kruskal–Wallis ANOVA on ranks with Dunn’s method from three experiments with 8–66 neurons per condition at each time point. LDH, Analysis on data from triplicates measured at each of four time points across three experiments using one-way ANOVA with Bonferroni correction for multiple comparison (^*^p < 0.05, ^∗∗^*p* < 0.01, ^∗∗∗^*p* < 0.005, and ^****^p < 0.0001).

Because the HD109 line demonstrated more pathotypes than the HD77 line, this more severe HD patient line was used in subsequent experiments and compared to the CTR33 line. As astrocytes promote neuronal development (Pfrieger and Barres, 1997; Pfrieger, 2010), the contribution of HD astrocytes to neuronal physiology was assessed by co-culturing *Stage 4* HD astrocytes with iPSC-derived striatal neurons (Telezhkin et al., 2016). To provide functional references, HD and control astrocytes and neurons were also analyzed alone in mono-cultures. Spontaneous intracellular Ca^2+^ waves are indirect measures of activity in astrocytes and synaptic firing in neurons, and so Ca^2+^ imaging was conducted after 6 days of co-culture. Neuronal events, observed in the small round cell bodies (Supplementary Figure 3A), were of short duration but involved a large change in fluorescent intensity from baseline levels (Figure 4A). Astrocytic transients, observed in the large cell bodies (Supplementary Figure 3B), were of significantly longer duration and had a smaller change in emission intensity (Figure 4B). To confirm the identity of these spontaneous events, TTX was used at the end of recordings to block the Na_*v*_-dependent neuronal action potentials (data not shown).

Co-culture with control or HD astrocytes led to a striking increase in the percent of neurons with spontaneous events compared to neurons cultured alone (*p* < 0.01; Figure 4C). In addition, the frequency of events increased when neurons were co-cultured with control and HD astrocytes (*p* < 0.001; Figure 4D). Of note, however, the co-cultured HD astrocytes were significantly less supportive of spontaneous events and the frequency of events in control neurons (*p* < 0.05; Figures 4C,D) compared to control astrocytes. This suggests that while both astrocytes are supportive of neurons, the HD astrocytes are less supportive of control neurons compared to control astrocytes.

When we examined Ca^2+^ waves in the iPSC-derived astrocytes, we saw an increase in the proportion of astrocytes exhibiting spontaneous transients when co-cultured with neurons (*p* < 0.05; Figure 4E). Although no difference was observed in the amount of transient activity in monoculture, the duration of transients in HD astrocytes was longer compared to control astrocytes (*p* < 0.05; Figure 4F). Further, co-culture with neurons decreased the duration of transients in both HD and control astrocytes (*p* < 0.001; Figure 4F), which suggests the neuron co-culture enhances the speed of intracellular calcium signaling within astrocytes. However, in co-culture with either control or HD neurons, the HD astrocytes generated longer events compared to those in control astrocyte co-cultures (*p* < 0.05; Figure 4F). This is a novel phenotype observed in HD astrocytes, which could be the result of impaired Ca^2+^ modulation in HD iPSC-derived astrocytes, similar to what has been reported in HD neurons (Lim et al., 2008).

To expand upon the Ca^2+^ imaging results and evaluate the effects of co-culture over time, we employed microelectrode array (MEA) analysis of *Stage 4* HD and control astrocytes co-cultured with HD and control neurons (Figure 4G). MEA allows for non-invasive recordings of extracellular action potentials generated by neuronal activity (Spira and Hai, 2013), which we quantified as the mean spike number per neuron over 5 min. First, we compared mono-culture and co-culture of HD and control astrocytes and neurons from days 9–13 (Figure 4H). As seen via calcium imaging, no difference was observed in the activity between HD and control neurons in mono-culture, while co-culture with astrocytes caused an increase in activity. In contrast with calcium imaging, we did not observe lower activity levels in neurons co-cultured with HD astrocytes. Instead, co-culture of HD astrocytes with either control or HD neurons caused an increase in spiking activity compared to that observed with control astrocytes (Neuro^CTR^: 48.49 ± 14.53, Neuro^HD^: 47.47 ± 24.50, Neuro^CTR^+Ast^CTR^: 63.12 ± 14.64, Neuro^HD^+Ast^CTR^: 68.89 ± 15.21, Neuro^CTR^+Ast^HD^: 96.43 ± 21.80, Neuro^HD^+Ast^HD^: 84.45 ± 17.37 spikes/5 min, *n* = 15 to 207 neurons per condition, three separate experiments; *p* < 0.001, *p* < 0.005, and *p* < 0.0001; Figure 4H). The contrast between calcium imaging data and MEA activity is likely due to the later time points of analysis in MEA (an additional 5 days in co-culture), which could allow for neurons to mature further. However, when the activity was observed over the entire 25-day duration, no differences between the HD and control astrocyte co-cultures could be seen (*n* = 8 to 166 neurons per time point, three separate experiments; *p* = 0.293; Figure 4I). This demonstrates that the initial decreased activity in control neurons that are co-cultured with HD astrocytes observed with calcium imaging, and then increase in activity of both control and HD neurons that are co-cultured with HD astrocytes, are transient phenotypes that attenuate over time in co-culture, as evident by the longer duration MEA recordings.

### HD Astrocytes Cannot Protect Against Glutamate-Mediated Neuronal Toxicity

Decreased glutamate re-uptake is a component of the dysfunction with striatal HD astrocytes (Bradford et al., 2009; Faideau et al., 2010; Khakh et al., 2017). As such, we next assessed glutamate-induced toxicity in astrocytes and co-cultures by measuring lactate dehydrogenase (LDH) activity in the culture medium after a 12-h incubation with excess (150 μM) glutamate (Figure 4K). When cultured alone, the HD astrocytes had higher levels of LDH activity compared to control astrocytes (Ast^CTR^: 0.037 ± 0.001, Ast^HD^: 0.070 ± 0.004 absorbance, *n* = 4 assays per each of three experiments; *p* < 0.001). Indeed, co-culture of control astrocytes with either HD or control neurons did not increase the levels of LDH activity over that of control astrocytes alone. This suggests that with control astrocyte co-culture, neurons of either genotype do not cause an increase in toxicity in response to glutamate. However, in the presence of HD astrocytes, toxicity levels were increased with co-culture of both control and HD neurons compared to control astrocyte co-cultures, respectively (Neuro^CTR^+Ast^CTR^: 0.047 ± 0.004, Neuro^HD^+Ast^CTR^: 0.060 ± 0.006, Neuro^CTR^+Ast^HD^: 0.094 ± 0.007, Neuro^HD^+Ast^HD^: 0.092 ± 0.007 absorbance; *p* < 0.005 vs. control neuron; *p* < 0.001 vs HD neuron; Figure 4K). Collectively, this data indicates that the HD astrocytes are less proficient in preventing glutamate-induced toxicity in neuron co-cultures compared to control astrocytes, regardless of the disease state of the neurons.

### GABA Expression Is Reduced in Neurons Co-cultured With HD Astrocytes Compared to Control Astrocytes

**FIGURE 5:**
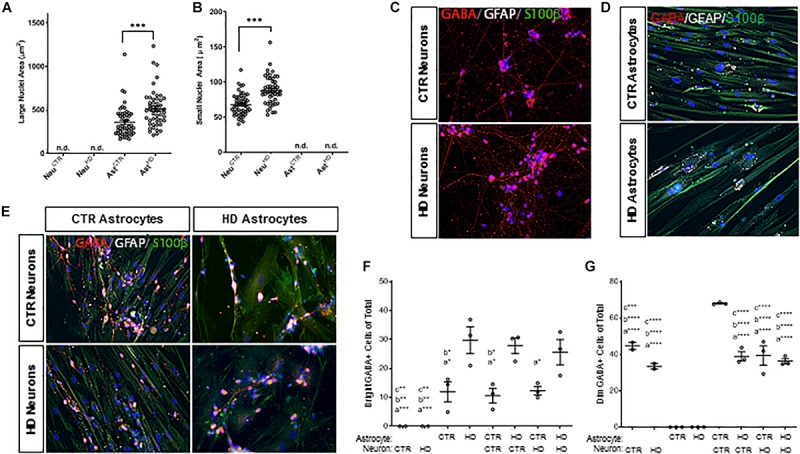
*Stage 4* HD (HD109) and control (CTR33) astrocytes (large nuclei, >210 μm^2^) and neurons (small nuclei, <141 μm^2^) in mono- or co-culture display differences in both nuclear size and percent of cells expressing GABA. **(A)** HD astrocyte nuclei are larger than control astrocytes. No large nuclei are detected in the neuronal cultures. **(B)** HD neuronal nuclei are larger than control neuronal nuclei. No small nuclei are detected in the *Stage 4* astrocyte cultures. **(C–E)** GABA (red), GFAP (white) and S100β (green) expression in panel **(C)** control and HD neurons, **(D)** control and HD astrocytes, **(E)** co-culture matrix of HD and control astrocytes/neurons. **(F)** HD astrocytes in mono-culture or co-culture have a higher percentage of cells expressing a dim, punctate GABA compared to control astrocytes. Significance between conditions denoted as “a”: from Ast^HD^, “b”: from Neu^CTR^ + Ast^HD^, “c”: from Ast^HD^ + Neu^HD^. **(G)** Control neurons in mono-culture have a higher percentage of cells expressing a bright cytoplasmic GABA stain than HD neurons in mono-culture, which is increased upon addition of control astrocyte co-culture. Significance between conditions denoted as “a”: from Ast^CTR^, “b”: from Ast^HD^, “c”: from Ast^CTR^ + Neu^CTR^. These cells were analyzed from more than three separate experiments. Ast, astrocyte; Neu, neuron; CTR, control. One-way ANOVA (^*^p < 0.05, ^∗∗^*p* < 0.01, ^∗∗∗^*p* < 0.001, and ^****^p < 0.0001).

An interesting observation of cells in mono-culture was that astrocytes had significantly larger nuclei (*p* < 0.0001; Figure 5A) compared to the smaller nuclei (Figure 5B) in iPSC-derived neurons, which was consistent in co-cultures (*p* < 0.0001; Supplementary Figure 4). This characteristic was used to subsequently discriminate between astrocytes and neurons. Additionally, both HD astrocytes (CTR Ast: 361.7 ± 24.93, HD Ast: 516.2 ± 29.89 μm^2^, *n* = 50 each; *p* < 0.0001; Figure 5) and neurons (CTR Neuro: 68.18 ± 2.22, HD Neuro: 88.44 ± 2.82 μm^2^, *n* = 50 each; *p* < 0.0001; Figure 5B) had significantly larger nuclei compared to the respective control cells.

The neurotransmitter GABA is traditionally a marker of inhibitory neurons (Molgaard et al., 2014), but it is also expressed in glia (Lee et al., 2011), and levels of expression are brain-region dependent (Yoon et al., 2011). Therefore, we first assessed whether GABA expression differed in HD and control lines. Immunocytochemistry showed that the cellular localization of GABA was completely different in neurons (small nuclei; Figures 5C,E) and astrocytes (large nuclei; Figures 5D,E). IPSC-derived neurons had classical cytoplasmic GABA expression in the form of dim puntca throughout the cytoplasm, whereas astrocytes mainly expressed GABA in bright perinuclear foci (Jo et al., 2014). We found that more HD astrocytes expressed GABA-positive perinuclear foci than control astrocytes, and this pattern was independent of culture state (Ast^CTR^: 11.86 ± 3.50, Ast^HD^: 29.72 ± 4.66, Ast^CTR^+Neuro^*CTR*^ 10.56 ± 2.55, Ast^CTR^+Neuro^HD^ 12.23 ± 1.45, Ast^HD^+Neuro^CTR^ 27.80 ± 2.69, Ast^HD^+Neuro^HD^ 25.59 ± 4.38 bright GABA+ cells of total, *n* = 50 cells each, three experiments; Figure 5F). Interestingly, while there was no difference in the percentage of HD or control GABA-positive neurons in mono-culture, the percentage of GABA-positive control neurons increased when co-cultured with control astrocytes (Neuro^CTR^: 44.63 ± 2.03, Neuro^HD^: 33.44 ± 1.53, Ast^CTR^+Neuro^CTR^ 67.98 ± 0.38, Ast^CTR^+Neuro^HD^ 39.35 ± 5.25, Ast^HD^+Neuro^CTR^ 38.94 ± 2.43, Ast^HD^+Neuro^HD^ 36.25 ± 1.42 dim GABA+ cells of total, *n* = 50 cells each, three experiments; Figure 5G). This supports reports that astrocyte co-culture is beneficial for neurogenesis and functional maturation (Johnson et al., 2007). However, this astrocyte-facilitated augmentation did not occur when HD astrocytes were cultured with either control or HD neurons, indicating that HD astrocytes are not as supportive of neuronal maturation as control astrocytes. Additionally, control neurons co-cultured with control astrocytes demonstrated increased GABA expression, but this increase was not observed in HD neurons, suggesting an inherent deficiency in HD neurons that cannot be rescued by control glia.

## Discussion

Here, we present data from a novel protocol to generate iPSC-derived astrocytes that are physiologically mature. Using this protocol, HD astrocytes recapitulate phenotypes previously seen in HD mouse models, such as impairment of K_*ir*_ currents, lengthened spontaneous Ca^2+^ waves, and reduced cell membrane capacitance. They also demonstrated that while the control astrocytes lead to increased neuronal maturation, HD astrocytes were poorly supportive of neuronal functional maturation. Overall, this study supports and extends the idea that there are non-cell autonomous contributions of astrocytes to the HD phenotype and shows that iPSC-derived astrocytes and neurons in co-culture can be used to effectively model HD.

### Electrophysiological Assessment of iPSC-Derived Astrocytes *in vitro*

Maturation of our astrocyte cultures was examined via electrophysiological assessment. As expected, the intrinsic membrane properties differed between immature (Type A and B) and mature (Type C and D) astrocytes, which validated a specification of maturation states (Higashi et al., 2001; Kofuji and Newman, 2004). As evident in primary rodent astrocytes, specific differences in intrinsic properties are observed in electrophysiologicaly distinct astrocyte populations of which the proportion of populations present changes as the animal ages (Kimelberg et al., 2000; Zhou et al., 2006). For example, astrocytes of the newborn rat hippocampus exhibit high membrane resistance and depolarized membrane potentials, which decrease and hyperpolarize with age, respectively. We observed a similar trend in properties measured in our iPSC-derived astrocyte Types. The astrocyte Types of our cultures were further validated by expression of specific K^+^ currents that are expressed by astrocytes *in vivo* across development. In the rat hippocampus, the most prevalent population of astrocytes in young animals (P1–P15) exhibit outward-rectifying, voltage-gated potassium currents, whereas the more mature astrocyte population observed in older animals (P8–P106) presented more ohmic features of passive potassium currents (Zhou et al., 2006). However, it is important to note that our mature Type D astrocytes reach a maturation profile comparable to intermediately mature astrocytes observed in rodent preparations. This is not surprising, as human iPSC-derived models have been shown to achieve maturation levels similar to that observed in human fetal tissue yet fall short of adult tissues based upon transcriptomic analysis (Ho et al., 2016). Still, the trend identified in our astrocyte Types that show maturation of intrinsic properties and expression of specific potassium currents indicate that regardless of disease state, Type C/D astrocytes were the most mature astrocytes with generally larger and more complex physiology.

### Glial Dysfunction in HD

Reactive astro-gliosis can be identified as an early feature of HD, due to the increased GFAP immunoreactivity in prodromal gene-positive patients that continues to increase with disease progression (Faideau et al., 2010). HD patient iPSC-derived astrocytes display autophagocytic vacuoles (Juopperi et al., 2012) and increased evoked inflammatory responses (Hsiao et al., 2014). In our study, the iPSC-derived HD astrocytes were found to have larger cell capacitances than control astrocytes, indicative of an increased cell size. This increase in astrocyte cell size is linked to reactive astrocytosis (Amenta et al., 1998). Additionally, larger nuclei have been previously observed in HD astrocytes, which has been attributed to a reactive astrocyte pathotype (Aschner and Kimelberg, 1996). This mirrors the larger HD astrocyte nuclei that was also observed in our study. Additionally, increased GABA expression observed in HD astrocytes has been linked to reactive astrocytosis (Jo et al., 2014), and, depolarized HD astrocytes have a reduced ability to release GABA via GAT3 (Wójtowicz et al., 2013). This could explain the accumulation of GABA seen in HD astrocytes in our study. Another explanation could be a reduced capability for HD astrocytes to properly catabolize GABA. It has been previously demonstrated that in HD animal astrocytes GABA-glutamine cycling is compromised (Skotte et al., 2018), and this could lead to an astrocytic accumulation of GABA. Together, these observations truly suggest that *in vitro* generation of HD iPSC-derived mature astrocytes generates a reactive astrocyte population similarly to that observed *in vivo.*

Astrocytes in HD mouse models have alterations in mechanisms involved in extracellular ionic homeostasis, including a decrease in expression of astrocyte-enriched K_*ir*_4.1 channels (Tong et al., 2014). This reduction in K_*ir*_ current contributes to dysfunctional astrocyte glutamate and Ca^2+^ signaling at a stage in mouse models which is similar to the prodromal stage in patients (Jiang et al., 2016). This dyshomeostasis of astrocytes contributes to the neuronal loss seen in disease, due to elevated extracellular K^+^ and the concomitant augmentation of neuronal excitability linked to neuronal toxicity in HD (Tong et al., 2014). In fact, overexpression of K_*ir*_4.1 channels in HD mouse astrocytes *in vivo* normalizes extracellular K^+^, reverses aspects of neuronal dysfunction, increases survival and attenuates some motor phenotypes. In the present study, the HD iPSC-derived Type C astrocytes displayed reduced peak K_*ir*_ current densities compared to control astrocytes, similar to the reduction seen *in vivo* (Tong et al., 2014). This K_*ir*_ current density reduction was not mirrored in either the Type A/B K_*v*_ or Type D K_2P_ current densities. It has been previously demonstrated that immature glial cells lack expression of K_*ir*_ channels (Sontheimer, 1994; Bordey and Sontheimer, 1997; MacFarlane and Sontheimer, 2000), which would explain the lack of phenotype in the Type A/B population. Upon astrocyte maturation, an increase in K_*ir*_ channel expression causes a hyperpolarization of RMP from -50 to -80 mV (Ransom and Sontheimer, 1995; Bordey and Sontheimer, 1997). This hyperpolarization is mirrored in the Type A/B transition to Type C/D astrocytes. Not surprisingly then, the depolarized resting membrane potential in Type C HD astrocytes coincides with the reduced K_*ir*_ current density. Similarly, the absence of a significant difference between HD and control astrocytes in K^+^ current density was consistent with a lack of difference in RMP in the Type D astrocytes, which display passive K_2P_ currents rather than the K_*ir*_ currents.

### Non-cell Autonomous Effects Seen in HD Using Glial-Neuronal Co-culture Paradigms

Non-cell autonomous effects of astrocytes in HD have been demonstrated. Control murine neurons co-cultured with HD glia are more susceptible to glutamate excitotoxicity (Shin et al., 2005). Conversely, control murine glia provide protection from excitotoxicity as seen in primary HD mouse neurons (Shin et al., 2005). The data presented here with human iPSC-derived astrocyte-neuron co-culture recapitulate those findings. When we co-cultured control iPSC-derived neurons with control iPSC-derived astrocytes, the neurons had an increased proportion of GABA-expressing cells and an increase in spontaneous activity at early stages of co-culture. These are indicative that control astrocytes support increased neuronal maturation more efficiently than neuronal mono-culture. This is not surprising, as the benefit of astrocyte co-culture for neuronal maturation and support is well established (Johnson et al., 2007). However, when control neurons were plated onto HD astrocytes for 6 days, there was no increase in neuronal GABA expression, and while there was increased spontaneous activity as compared to in mono-culture, it was markedly less than co-culture with control astrocytes. In agreement with the delayed development phenotype previously proposed for HD iPSC-derived models (Mattis et al., 2015; Ring et al., 2015; HD iPSC Consortium, 2017), the HD and control neurons co-cultured with HD astrocytes did achieve similar firing rates after extended time in co-culture. Additionally, co-cultures of either control or HD neurons with HD astrocytes demonstrated increased toxicity after chronic glutamate exposure, as compared to neurons on control astrocytes. These observations demonstrate that the HD astrocytes do not support neuronal maturation or function as effectively as control astrocytes. This agrees with previous data demonstrating that HD murine glia have a reduced ability to support neurons due to a reduced ability to remove extracellular glutamate, which contributes to the neuronal excitotoxic pathotype in HD (Shin et al., 2005).

In this study, we generated physiologically mature iPSC-derived astrocytes from HD patients and control subjects. More excitingly, these astrocytes recapitulated an important astrocyte HD pathology previously measured in mouse models, the impairment of K_*ir*_ currents, which had not yet been measured in humans. Furthermore, we employed this novel, iPSC-based human cell model to identify additional astrocyte HD-pathotypes, including lengthened spontaneous Ca^2+^ waves and reduced cell membrane capacitance. In the future, this human HD patient iPSC-based neuronal-glial co-culture system can be used to recapitulate key aspects of the disease for further elucidation of mechanistic pathways and therapeutic intervention.

## Data Availability

All datasets generated for this study are included in the manuscript and/or the Supplementary Files.

## Ethics Statement

All procedures were performed in accordance with the IRB guidelines at the Cedars-Sinai Medical Center under the auspice IRB-SCRO Protocols: Pro00028429, Pro00021505, and Pro00032834.

## Author Contributions

CS, PK, NA, VG, DR, and VM designed the study. DR, VG, and CT carried out the study. DR, VG, and VM analyzed the data. VG and VM wrote the manuscript.

## Conflict of Interest Statement

The authors declare that the research was conducted in the absence of any commercial or financial relationships that could be construed as a potential conflict of interest.
