# Supplementary material for: Huntington’s Disease Patient-Derived Astrocytes Display Electrophysiological Impairments and Reduced Neuronal Support
Source: Front Neurosci. 2019 Jun 28;13:669. doi: 10.3389/fnins.2019.00669 (PMC6610155; doi:10.3389/fnins.2019.00669)
Supplement: Supplementary file 1 [file Data_Sheet_1.PDF]

Supplementary Figure 1

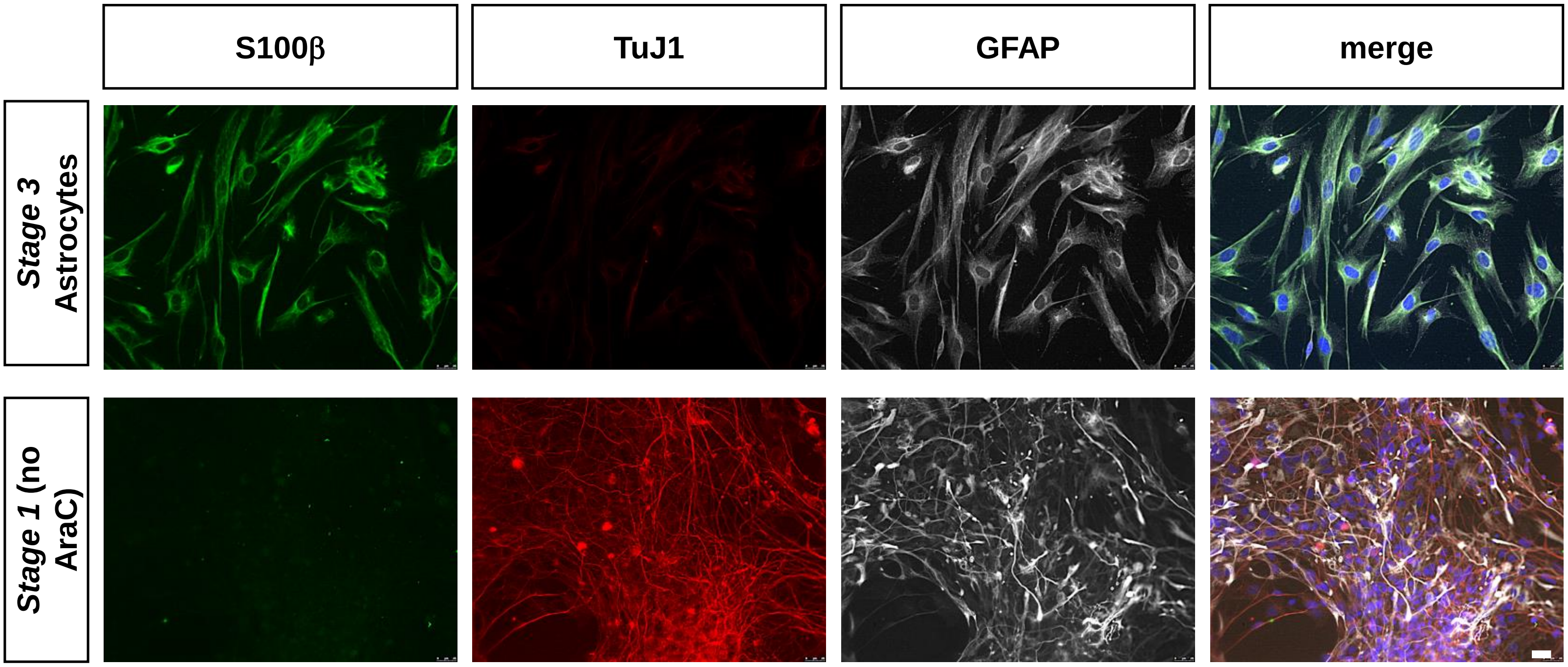

Supplementary Figure 1. *Stage 3* iPSC-derived astrocytes are positive for S100β and GFAP, but not TuJ1. After one week in *Stage 3* media (left panel; 3 weeks total of glial differentiation), the cells stained positive for glial markers S100β (green) and GFAP (grey), but were not positive for the neuronal marker TuJ1 (red). They also had a more typical glial morphology, with large, flattened cell bodies. In contrast if cells are left for the same duration (3 weeks) in *Stage 1* media without Ara C treatment, the cells spontaneously differentiate into immature GFAP-positive, S100β-negative glia and TuJ1-positive neurons, with a typical neural progenitor morphology. The blue color in the merged panels is a DAPI nuclear stain.

Supplementary Figure 2

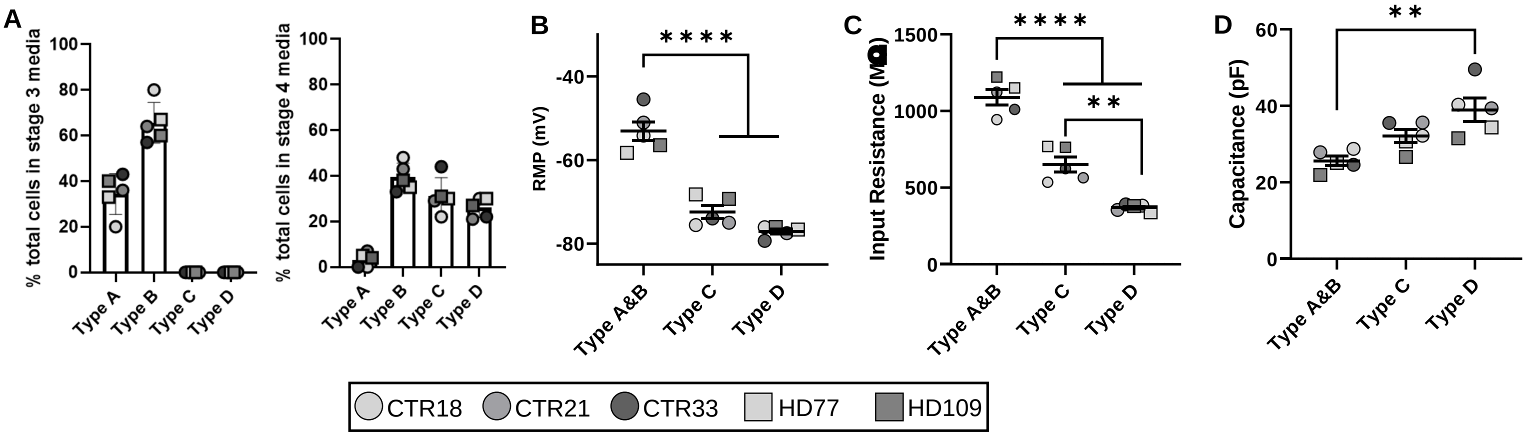

Supplementary Figure 2. Changes in passive membrane properties between astrocyte Types indicates changes in maturation states. (A) Proportion of astrocyte maturation Types observed in stage 3 (left) and stage 4 (right) media. (B) Mean resting potential decreased from Types A/B to C to D. (C) Input resistance also decreased from Types A/B to C to D. (D) Whole cell capacitance increased from Type A/B to Type D. Each circle represents the average of a line (n= at least three experiments) of a certain type, with five lines shown per Type. (one-way ANOVA with Bonferroni multiple comparison; ** p <0.01, ****p < 0.0001)

Supplementary Figure 3

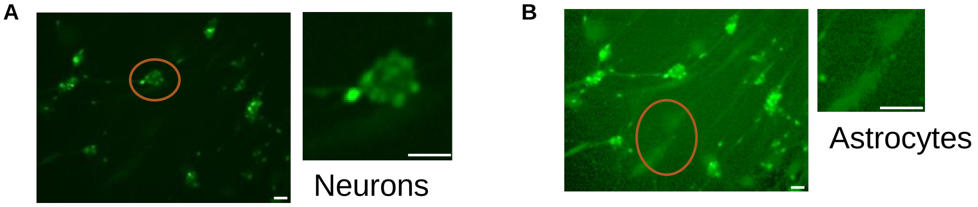

Supplementary Figure 3. Calcium transients in co-cultures of HD (HD109) and control (CTR33) astrocytes and neurons demonstrate alteration in neuronal firing. (A) Images of calcium loading in neurons (green). (B) Images of calcium loading in astrocytes (green). Scale bar 50 µm.

Supplementary Figure 4

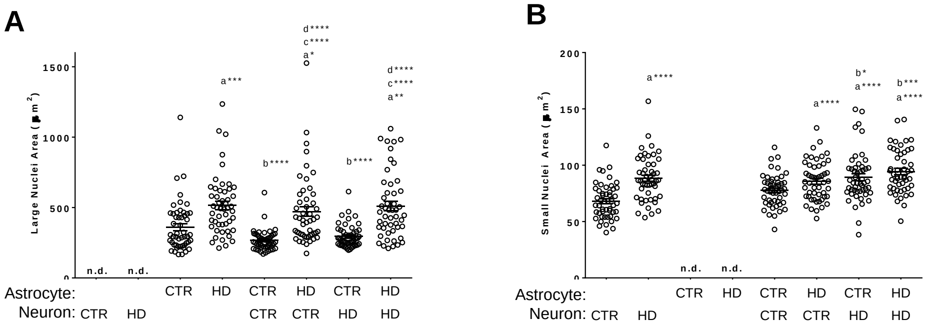

Supplementary Figure 4. *Stage 4* HD (HD109) and control (CTR33) astrocytes (large nuclei, > 210 µm^2) and neurons (small nuclei, < 141 µm^2) in mono- or co-culture display differences in nuclear size. (A) HD astrocyte nuclei are larger than control astrocytes in mono- and co-culture, but co-culture with control neurons results in smaller HD astrocyte nuclei. Significance between conditions denoted as “a”: from Ast^{CTR}, “b”: from Ast^{HD}, “c”: from Ast^{CTR} + Neu^{CTR}, “d”: from Ast^{CTR} + Neu^{HD}. (B) HD neuronal nuclei are larger than control neuronal nuclei, with or without co-culture. However, control neurons co-cultured with HD astrocytes have larger nuclei. Significance between conditions denoted as “a”: from Neu^{CTR}, “b”: from Neu^{HD}. (Abbreviations: astrocyte (Ast), neuron (Neu), control (CTR); One-way ANOVA; *p < 0.05, **p <0.01, ***p < 0.001, ****p < 0.0001)
